# Models of Concurrent Disorder Service: Policy, Coordination, and Access to Care

**DOI:** 10.3389/fpsyt.2019.00061

**Published:** 2019-02-19

**Authors:** Mary Wiktorowicz, Aber Abdulle, Kaitlin Di Pierdomenico, Sheila A. Boamah

**Affiliations:** ^1^School of Health Policy and Management, York University, Toronto, ON, Canada; ^2^Faculty of Nursing, University of Windsor, Windsor, ON, Canada

**Keywords:** concurrent disorders, mental health and substance use, substance use disorders, mental health and addictions, substance misuse, substance use, mental health policy

## Abstract

**Background:** Societal capacity to address the service needs of persons with concurrent mental health and substance-use disorders has historically been challenging given a traditionally siloed approach to mental health and substance-use care. As different approaches to care for persons with concurrent disorders emerge, a limited understanding of current models prevails. The goal of this paper is to explore these challenges along with promising models of coordinated care across Canadian provinces.

**Materials and methods:** A scoping review of policies, service coordination and access issues was undertaken involving a review of the formal and gray literature from 2000 to 2018. The scoping review was triangulated by an analysis of provincial auditor general reports.

**Results:** Models of concurrent disorders service were found to have evolved unevenly. Challenges related to the implementation of models of collaborative care and local networks that foster service coordination and policy accountability were found to inhibit integrated care.

**Conclusion:** Emergent models of coordinated care were found to include collaborative care, regional networks with centralized access to care, clinical information-sharing, cross-training, improved scope of care to include psychologists and alignment of physician incentives with patient needs to better support patient care.

## Introduction

Co-occurring mental health and substance-use disorders disable one-third to one-half of treatment populations with an estimated international prevalence of 1–3% (1, 2). A large proportion of individuals with concurrent disorders report high levels of unmet need and low levels of satisfaction with care (3). As many as 35–50% of those with concurrent disorders do not access formal care. Concurrent disorders offer unique challenges for healthcare providers, as substance use may affect adherence to treatment or compromise efficacy of prescribed medications (4). Concurrent disorders are associated with higher levels of service use when compared with either substance-use or mental illness alone. Individuals with concurrent disorders are three to four times more likely to be hospitalized than those with only mental illness, and are 10–20 times more likely to be admitted to inpatient care than those with substance-use disorders alone (5). Individuals with concurrent disorders have a greater tendency to miss medical appointments, experience relapse, and be readmitted to hospital than individuals with only mental illness (6, 7). They are also more likely to experience higher rates of morbidity, mortality, unemployment, poverty, homelessness, social isolation (2, 8, 9) and involvement with the criminal justice system (10) than those singly diagnosed (2) leading to immense health, social and economic costs (8, 9, 11).

The Canadian population affected by concurrent disorders (1.7%) accounts for a large proportion of those using mental health and substance use (MHSU) services (12). Concurrent disorders are common within substance-use subpopulations (8). As post-traumatic stress disorder and use of opioids rise, so has the prevalence of concurrent disorders (13) and with it the imperative to develop effective models of care. Over the years, consensus on models of integrated MHSU care has been slow to evolve. Integrated treatment can be differentiated from sequential and parallel treatment, with integrated approaches largely preferred. Sequential treatment has been criticized for ignoring the interconnected nature of concurrent disorders, and parallel approaches can lead to contradictory or incompatible treatment and inferior outcomes (14, 15). Given the challenges associated with developing integrated treatment approaches, the objective of our study was to identify similarities and differences in emergent models of concurrent disorders service across Canadian provinces through a scoping review. Based on our findings, we aim to shed light on current service models, clarify the barriers that prevent the needs of affected individuals from being met and the changes needed at provincial and national levels to address the holistic needs of persons with concurrent disorders.

## Methods

### Search Strategy and Selection Criteria

We systematically reviewed the published literature available from 2000 to 2018 to identify relevant studies on concurrent disorders service policy, coordination and access to care to conduct a scoping review. Literature searches were carried out in the following electronic databases: MEDLINE, EMBASE, PsycINFO, Global Health, The Cochrane Library (Cochrane Database of Systematic Reviews, Cochrane Central Register of Controlled Trials (CENTRAL), Cochrane Methodology Register), Health Technology Assessment Database, and Web of Science (Science and Social Science Citation Index). Search terms included substance use/misuse/abuse, drug addiction, addiction therapy, substance use/misuse/abuse therapy, mental health services supports/treatment, concurrent disorders/mental health/substance use, substance use policy, concurrent disorder policy, concurrent disorder financing/funding, access to services, comorbidity. The search terms were used in combination with the Boolean operators AND, OR, and ^*^(asterisk). We screened all the publications for eligibility based on relevance by reviewing the title and abstract. We included qualitative and quantitative studies focused on Canada as well as gray literature, commentary, proposals and editorials in the Canadian context. The language of publication was limited to English for reasonable analysis purposes. Studies in which participants were elderly people over 75 years of age were excluded. The PRISMA-P criteria for reporting a scoping review protocol was followed (16). In addition, provincial Auditor General Reports that monitor and evaluate MHSU care from 2012 to 2018 were analyzed to triangulate the scoping review findings.

### Data Abstraction

Data were recorded based on participant characteristics (e.g., whether participants had concurrent disorders), addressed access to concurrent disorder service support/treatment programs, coordination of MHSU care and policy. Study quality was assessed using a checklist and included assessment of control group and randomization (for intervention studies), objectivity of outcome measures, validity, adequate methods of analysis (for qualitative studies), and description of the demographic.

## Results

**Figure 1:**
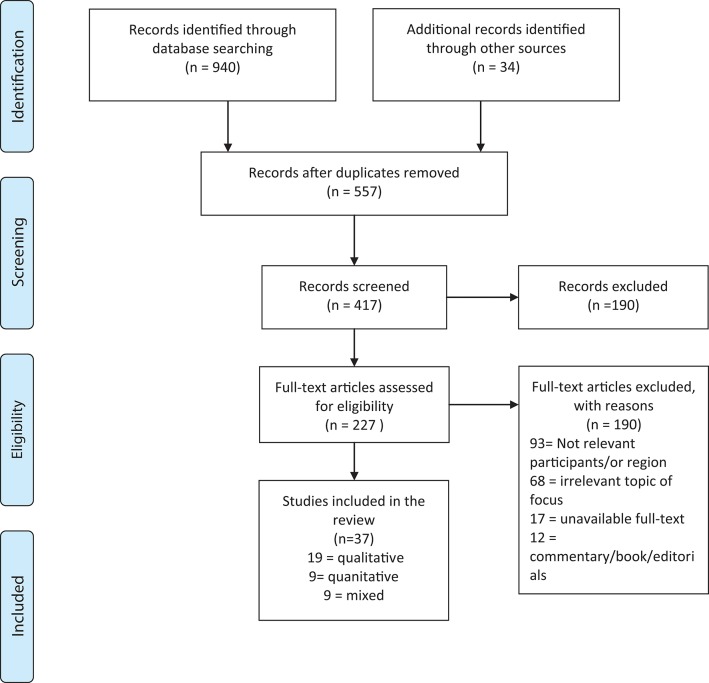
Search, screening, selection, and inclusion process diagram (16).

The screening and selection process for the scoping review are shown in Figure 1. Initially, the search identified 940 possibly relevant papers. Approximately 34 additional records were identified by scanning the references of the 940 included studies. In total, 557 papers were excluded due to repetition, which left 417 articles to screen. Once the title and abstract of each paper was scanned, 190 papers were omitted due to irrelevance in terms of the inclusion criteria. Of the remaining 227 papers, 190 were discounted due to either region (e.g., United States), demographic (e.g., patients with dementia), irrelevant topic (e.g., comparing treatments), unavailable full text, dissertation or book. The remaining 37 studies met inclusion criteria and were included in the final review: 19 qualitative (17–35), 9 quantitative (36–44), and 9 mixed methods articles (45–53) (Table 1). Reoccurring themes included access to care (strategies to improve access, community level of care and families, homelessness); integration and coordination of care (scope of practice for psychiatrists/physicians, access to and coordination with psychotherapy); and gaps in care. Lastly, in studies focused on policy, sub-themes of accountability, monitoring, and funding for initiatives and programs that address concurrent disorders emerged. The scoping review results were triangulated with an analysis of 13 auditor general reports that evaluated and monitored provincial MHSU programs and were incorporated in the policy section on accountability and monitoring.

**Table 1:** Summary of scoping review studies.

**Reference #**	**Study aim**	**Methods**	**Sample size**	**Year**	**Key findings**
McMain and Ellery (17)	Reviews the psychometrics of instruments for screening and diagnosis of personality disorders (PDs), which may be useful in addiction treatment settings.	Diagnostic assessment involving screening for personality pathology for people seeking treatment for addiction problems	N/A	2008	The prevalence of PDs among people with a SUD is high, and the clinical presentation of these patients is often more complex than that of their non-PD counterpart.
Séguin et al. (18)	Investigates all suicide cases in New Brunswick from April 1, 2002, to May 31, 2003 (14 months), to determine 6-month and lifetime prevalence rates of psychopathology in the deceased.	Direct proxy-based interviews and medical chart reviews, together with telephone contacts with informants.	*N* = 102	2006	At time of death, 65% of the suicide victims had a mood disorder, 59% had a SUD, and 42% had a concurrent mood and substance use disorder. The lifetime prevalence of SUDs among these suicide victims was 66%. Finally, 52% of the suicide victims presented with a personality disorder; one-half of these were of the cluster B type.
Fleury et al. (19)	Aims to identify integration strategies implemented in Quebec substance-use disorder networks and to assess their strengths and limitations.	A total of 105 stakeholders representing two regions and four local substance-use disorder networks participated in focus groups or individual interviews.	65 clinicians and 40 managers	2016	Six types of service integration strategies were implemented to varying degrees in substance-use disorder networks. They are: (1) coordination activities-governance, (2) primary-care consolidation models, (3) information and monitoring management tools, (4) service coordination strategies, (5) clinical evaluation tools and (6) training activities
Hunsley (20)	Reviews on cost issues associated with psychological interventions, including cost effectiveness and cost offset.	A review of the current fiscal situation in Canada as it relates to health care costs in general and psychological services more specifically.	N/A	2003	Psychological treatments (i) can be cost-effective forms of treatment and (ii) have the potential to reduce health care costs, as successfully treated patients typically reduce their use of healthcare services
Selick and Wiktorowicz (21)	Investigates the state of service integration in Ontario and identifies models for integrated treatment, factors that support or hinder implementation efforts.	Key informant interviews. Interview transcripts were analyzed to identify emerging themes.	N/A	2016	Five domains were identified: organizational barriers, system barriers, historical barriers, barriers related to stigma and discrimination, and knowledge barriers.
Wiktorowicz et al. (22)	Modes of governance were compared in 10 local mental health networks in rural/urban and regionalized/ non-regionalized contexts to clarify the governance processes that foster inter-organizational collaboration and the conditions that support them.	Case studies of 10 local mental health networks were developed using qualitative methods of document review, semi-structured interviews and focus groups that incorporated provincial policy, network and organizational levels of analysis.	*N* = 10 networks; 96 key informants (managers, clinicians)	2010	Mental health networks adopted either a *corporate structure, mutual adjustment* or an *alliance* governance model. Mediation by a regional authority was an important lever to foster formal inter-organizational coordination.
Brousselle et al. (23)	Identified key factors in integrating services for patients with co-occurring disorders.	A process evaluation with the aim of identifying factors that enhance or impede service integration.	N/A	2010	The study identified various levers and characteristics that affect the development of an integrated approach. Also formulated six propositions to identify what matters when integrating services for persons with mental health and substance use disorders.
Kêdoté et al. (24)	Described the characteristics of service utilization among patients with co- occurring disorders in a large urban area.	A sample of those identified with a SUD and psychoses from administrative and clinical databases were followed (12 months) to track their medical service use. A descriptive analysis of the data and a two-step cluster analysis were undertaken.	*N* = 5,467	2008	The analyses revealed relatively high utilization of emergency services, outpatient clinics, private practices, and hospitalization among patients with co- occurring disorders of severe mental illness and substance use.
Hunsley et al. (25)	To understand the complexity of the context in which psychological services are provided to young people.	Canadian psychological practitioners who offer services to children and youth were surveyed using real-time sampling to obtain a profile of services offered to a specific child or adolescent client.	*N* = 137	2014	In the majority of cases, psychological services involved not only the target client, but also parents or school personnel. Almost one third of clients had been prescribed psychotropic medication, and one quarter of practitioners indicated that their clients received services from another health care practitioner for the same problem.
Talbot et al. (26)	A survey among Anglophone and Francophone physicians in New Brunswick to determine practice and referral opinions to access specialized treatment services for adults with anxiety and depression.	The CPTADS is a 25-item self-report questionnaire to assesses demographics, practice characteristics, treatment approach to anxiety and depression, referral patterns, barriers to evidence-based practice, satisfaction with wait time and effectiveness of available treatment	*N* = 152	2014	The current findings suggest that many patients treated in primary care will not make it beyond their family physician's office and therefore will not access specialized therapy.
Fleury et al. (27)	Examines patient profiles in primary mental healthcare, determinants of service utilization, and primary mental healthcare reforms with a spotlight on best practices. Considers the most effective strategies for enhancing care collaboration and integration.	Conducted a major literature review, including both epidemiological and organizational research initiatives. General practitioner data from Quebec presented were sourced from two studies.	N/A	2012	Found that general practitioners welcomed opportunities to manage patients with common mental disorder; however, they also faced a number of obstacles, including: healthcare system fragmentation; lack of communication, resources, and clinical tools; the prevalence of solo practice; and unsuitable modes of payment.
Goldner et al. (28)	To obtain improved quality information regarding psychiatrist waiting times by use of a novel methodological approach in which accessibility and wait times are determined by a real-time patient referral procedure.	A semi structured call procedure was used to collect information about the psychiatrists' availability for receipt of referrals, identify factors that affect psychiatrist accessibility, and determine the availability of cognitive-behavioral therapy.	*N* = 297	2011	Among the 230 psychiatrists reached successfully and contacted, 160 (70%) indicated that they were unable to accept the referral.
McKee (29)	This review outlines the main best-practice guidelines for working with people with concurrent disorders and describes some of the barriers and facilitators to integration.	An example of successful integrated treatment is presented, with suggestions for how psychologists can play a key role in this important work.	N/A	2017	The change leader needs to be embedded within the program and remain long after initial integration to provide ongoing clinical supervision and model the novel, unified treatment philosophy.
Settipani et al. (30)	Identifies: (1) populations, settings, service providers, interventions, infrastructure and care coordination methods used in integrated care for youth with mental health and/or addiction needs; and (2) constructs measured and evaluated (e.g., outcomes, engagement) in youth integrated care.	Scoping review; a formal data extraction method was employed, enabling synthesis of results in quantitative and qualitative formats.	Seven electronic databases and gray literature sources were searched from 2001 to 2016	2017	The current focus of implementation efforts for youth integrated care in terms of the populations, settings, service providers, interventions, infrastructure and care coordination methods are outlined.
Durbin et al. (31)	Identifies funding arrangements and legislation/regulation related to scope of practice as important system wide factors that influence delivery of IC (Integrated care).	An environmental scan of scholarly literature using Ovid Medline, Embase and Social Work Abstracts and Google Scholar.	N/A	2016	Regarding the impact of funding, identified studies addressed patient selection, inclusion of non-physician providers (NPPs), and reimbursement for collaboration. Regarding regulatory/legal issues, identified studies addressed scope of practice for NPPs related to medication prescription and counseling, and the role of the physician.
Kates et al. (32)	A position paper which acknowledges that effective collaboration can involve providers from any discipline.	Focuses mainly on activities of family physicians and psychiatrists	N/A	2011	Recommends steps to enable MH&A services and primary care providers to work together to better meet the needs of populations that have difficulty gaining access to care they require, patient- centered style of practice, and influence the evolution of health care delivery in Canada.
Vallerand and McLennan (33)	Describes strategies of child mental health agencies to manage service demands; (2) determines whether strategies used are related to meeting Canadian Psychiatric Association (CPA) benchmarks and wait times; and, (3) determines whether strategies used are related to agency characteristics.	An online questionnaire distributed to agencies providing child mental health services in Canada. The survey inquired about agency characteristics, wait times, ability to meet benchmarks and a series of strategies which may impact wait times	*N* = 379	2013	One hundred thirteen agencies returned adequately completed surveys (29.8%). Collaborating with other agencies/providers and referring families to self-help resources were the most commonly endorsed strategies
Kozloff et al. (34)	Examines care and aftercare following first ED visit for psychotic disorder among youth.	A retrospective cohort study of first ED presentations for psychotic disorder among youth 16 to 24 years in Ontario, Canada.	*N* = 2,875	2018	Forty percent of youth discharged to the community from their first ED presentation for psychotic disorder received no outpatient mental health care within 30 days.
Nolin et al. (35)	In the absence of national standards, examines the current state of EIS (Early intervention services) for psychosis in Canada in relation to expert recommendations.	A detailed online benchmark survey was developed and administered to 11 Canadian academic EIS programs covering administrative, clinical, education, and research domains.	N/A	2016	Most surveyed programs offer similar services, in line with published expert recommendations. However, differences were observed in admission and discharge criteria, services for patients at ultra high risk (UHR) for psychosis, patient to clinician ratios, accessibility of services, and presence of specific inpatient units.
Latimer et al. (36)	Estimates average annual costs of homelessness by cost category, that homeless people with mental illness engender from the perspective of society.	990 participants were followed in 5 cities from 2009 to 2011 for up to 2 years. Questionnaires ascertained service use, income; city- specific unit costs were estimated.	*N* = 937 with useable data	2017	Net costs ranged from $C15,530 to $C341,535. Distribution of costs across categories varied significantly across cities. Lower functioning and a history of psychiatric hospital stays were the most important predictors of higher costs.
Fleury et al. (37)	Sought to identify factors associated with health service use by individuals with mental disorders in a Canadian catchment area.	Data was collected randomly from June to December 2009 by specially trained interviewers. A comprehensive set of variables was studied using Andersen's behavioral health service model. Univariate, bivariate, and multivariate analyses were carried out.	*N* = 406	2012	Emotional problems and a history of violence victimization were strongly associated with service use. Participants who were middle-aged or deemed their mental health to be poor were also more likely to seek mental healthcare. Individuals living in neighborhoods where rental accommodations were the norm used significantly fewer health services than those residing in neighborhoods where homeownership was preponderant; males were less likely to use services than females.
Kurdyak et al. (38)	To study the relationships among psychiatrist supply, practice patterns, and access to psychiatrists in Ontario Local Health Integration Networks.	Practice patterns of full-time psychiatrists and post discharge care to patients who were hospitalized for psychiatric care were analyzed, according to LHIN psychiatrist supply in 2009.	N = 1379	2014	As the supply of psychiatrists increased, out- patient panel size for full-time psychiatrists decreased, with Toronto psychiatrists having 58% smaller outpatient panels and seeing 57% fewer new outpatients relative to LHINs with the lowest psychiatrist supply.
Vasiliadis et al. (39)	Aimed to provide prevalence rates of health care service use for MH reasons by province and according to service type and to examine determinants of MH service use in Canada and across provinces.	Prevalence rate of past-year health service use for MH reasons, and potential determinants were assessed cross-sectionally, using Statistics Canada Canadian Community Health Survey: Mental Health and Well-Being data.	*N* = 36,984 respondents	2005	Need remains the strongest predictor of use, especially when a mental disorder is present. Barriers to access, such as income, were not identified in all provinces.
Torchalla et al. (40)	Examined the evidence of psychotherapeutic integrated treatment (IT) programs for individuals with concurrent substance use disorders and trauma histories.	Electronic searches of Cochrane Central Register of Controlled Trials, MEDLINE, Web of knowledge, PubMed, PsycINFO, CINAHL, PILOTS, and EMBASE identified 17 IT trials (9 controlled trials).	N/A	2012	Both narrative review and meta-analysis indicate that IT effectively reduces trauma symptoms and substance abuse from pretreatment to longest follow-up.
Denomme et al. (41)	Assessed the efficacy of a treatment program at reducing stress, increasing perceived social support from family and friends, and increasing general, dyadic, and self-rated family functioning within these concerned family members	A sample of family members of individuals with concurrent disorders was recruited, of which 97 participated in the treatment program and 28 were used as the comparison group.	*N* = 125	2017	A perceived personal benefits questionnaire demonstrated that participants had a better understanding of concurrent disorders, adopted stronger coping methods, participated in more leisure activities, and improved their relationship with the individual with a concurrent disorder.
Henderson et al. (42)	A protocol designed to test the benefits of an Integrated Collaborative Care Team (ICCT) model for youth with MHA challenges.	Youth presenting for hospital-based, outpatient psychiatric service will be randomized to ICCT or usual hospital-based treatment, using a pragmatic RCT.	*N* = 500	2017	First RCT of an ICCT program internationally. If equivalent clinical outcomes can be achieved with less expensive services, savings to the healthcare system may result.
Fleury et al. (43)	Identified variables associated with perceived unmet need for information, medication, and counseling, and overall perceived unmet needs related to mental health in a Montreal catchment area.	Needs were measured with the Perceived Need for Care Questionnaire and a comprehensive set of independent variables based on Andersen's behavioral model.	Of 2,334 persons interviewed 571 (24%) expressed a need	2015	Need factors were more strongly associated with unmet need for medication, predisposing factors with unmet needs for information and medication, and health service use with unmet information and counseling needs. People whose overall needs went unmet tended to be younger, to have an addiction, and to have consulted fewer professionals.
Durbin et al. (44)	Examined factors associated with unmet need for care from primary care physicians or from psychiatrists among clients enrolled in mental health court support programs in Toronto.	Cross-sectional study; sample included adults admitted to these programs during 2009 (*N* = 994). Predictors included client predisposing, clinical, and enabling variables.	*N* = 994	2014	Twelve percent had unmet need for care from primary care physicians and 34% from psychiatrists. Both measures of unmet need were associated with having an unknown diagnosis, having no income source or receiving welfare, homelessness, and not having a case manager.
Bartram and Lurie (45)	Explores how the gap in mental health funding occurred in Canada and provides a detailed analysis of the size of the gap itself.	Overview of provincial/territorial contributions, accountability mechanisms, outcome measures, the insurance/financing model, and how tightly eligible expenses are tied to specific initiatives, population groups, or levels of evidence.	N/A	2017	A public insurance-based funding model for psychotherapy and medication services are advised, but may not garner enough support given concerns with maintaining control over expenditures. However, the basket of mental health services is being examined by the Commissaire a la sante au bien etre in Quebec.
Rush and Saini (46)	Assesses and describes coordinated and centralized access for mental health and addictions in Ontario. Includes an assessment of what is being implemented, as well as what is being, or has been, planned and considered.	Relevant peer reviewed journal articles, reports and government documents published in English were searched from 1990 to 2015 using the search terms: “centralized access,” “centralized services,” “integrated care,” “coordinated care” etc. in Ontario	N/A	2016	Approaches to coordinated or centralized access have grown rapidly across the province. Many have appeared recently, and more are being developed. There is no published description of the different coordinated or centralized access approaches across Ontario. There also is no summary of research that can help improve and evaluate current approaches.
Vasiliadis et al. (47)	Compared the prevalence of depression and the determinants of mental health service use in Canada and the United States.	Data from preliminary analyses of the 2003 Joint Canada/US Survey of Health, which measured Canadian and American resident ratings of health and health care services. Included multivariate analysis of depression.	*N* = 3,505 Canadians; *N* = 5,183 Americans	2007	There was no difference in the prevalence of depression and mental health service use between Canadians and Americans with health insurance. Among those with depression, however, disparities in treatment seeking were found to be associated with lacking health insurance coverage in the US.
Cheung et al. (48)	Sought to understand correlation between ED use, hospital admission, and substance dependence among homeless persons with concurrent mental illness in a ‘Housing First’ (HF) intervention trial.	Two randomized controlled trials addressing homeless individuals with mental disorders who have “high” or “moderate” levels of need.	*N* = 497	2015	Substance dependence was not independently associated with ED use or hospital admission among homeless adults with mental disorders participating in an HF trial.
Roberge et al. (49)	(a) to examine access to psychotherapy for anxiety disorders in a sample of primary care patients; and (b) to examine individual factors associated with access to psychotherapy.	Data was drawn from the “Dialogue” project, a large primary care study conducted in 67 primary care clinics.	*N* = 740	2014	Nearly half of the respondents with anxiety disorders had received a form of psychotherapy or counseling in the past 12 months, and 20% of respondents reported at least 12 sessions with the same health care professional
Bradley and Drapeau (50)	Documents the attitudes of psychologists and psychotherapists licensed to practice in Quebec toward access to psychotherapy and government-funded psychotherapy programs.	Participants completed an online questionnaire; results indicated that 77% of the sample strongly agreed that accessibility to psychotherapy should be increased.	*N* = 1,275	2014	There was stronger agreement that clinicians working within a government-funded psychotherapy program should be paid on a session-to- session basis as opposed to receiving a yearly salary; to be able to set their own fee; and to have freedom to choose the appropriate psychotherapeutic approach (e.g., cognitive behavioral therapy [CBT], emotion-focused therapy [EFT]) and appropriate treatment materials (e.g., psychoeducational handouts).
Fleury et al. (51)	Evaluates the implementation and impact of a pilot project aimed at establishing an integrated service network for adults with severe mental disorders in an urban area in Quebec.	A case study method using formative assessment of a project designed to provide, through support for decision-making, ongoing information and results with regard to the project's ability to solve problems as they arise.	N/A	2008	This study shows that Integrated Service Networks play a role in transforming the health care system based on its existing structures and resources, allowing for a gradual transformation of the organization of services.
Dewa et al. (52)	Examines the changes in continuity of care (COC) likely to be affected by new system investments and the contributing factors.	A mixed method approach was used: decision-makers participated in two qualitative interviews; a 3-year cross-sectional quantitative data collection approach was used with clients and case managers.	*N* = 67	2010	A main finding was that new system investments can improve COC in terms of increased care access. However, it is not clear how other COC dimensions will be affected
Fleury et al. (53)	Assessed predictors and changes in adequacy of help received (AHR), as perceived by 204 individuals with severe mental disorders (SMDs) transferred from a mental health institution to the community following a key healthcare reform.	Assessed changes in perceived AHR among 204 persons with SMDs at three points in time: before the mental healthcare reform (T0), and at 2 years (T1) and 5 years (T2) after implementation of the reform.	*N* = 352	2016	The results confirm that patient transfers from the institution to the community as mandated in the Quebec Mental Health Action Plan produced positive short- term effects. Indeed, after 2-year follow-up (T1), adjusted perceived AHR remained stable.

### Access to Care

Considerable emphasis was placed on improving access to MHSU services across the provinces. Rush and Saini (46) identified five dimensions of access to care: approachability; acceptability; availability and accommodation; affordability; and appropriateness. The potential barriers to healthcare access are compounded by the social determinants (e.g., education, race, sex, ethnicity, and income). Patients further noted such barriers as stigma, low income, language differences, lack of integration between mental health and health services, shortage of mental health professionals, regional disparities, and cross-cultural diversity (46). Issues related to MHSU care were found to go well-beyond enabling service access when the most basic needs went unmet for vulnerable segments of the population.

Strengthening mental health care within primary care and integrating care between providers are key issues (27). Integrated service models provide a single-entry point to access a variety of services by coordinating medical care with allied community healthcare and social services. Although integrated service models are endorsed for improving access, efficiency, and quality of care, various barriers exist due to lack of coordination. In Canada, 60% of general practitioners are in private physician-run clinics, and a minority (8.3%) work within public governance models such as community health centers (54). From a collaborative perspective, 23% work in solo practice, 51% in group practices, and 24% in multidisciplinary team practices (27, 54).

#### Strategies to Improve Access

Although the notion of coordinated and integrated care is emphasized in the literature, the important shift is in the implementation of programs. Rush and Saini (46) highlight recent centralized programs available. Government policy reports such as *Open Minds, Healthy Minds, Ontario's Comprehensive Mental Health and Addiction Strategy* (2011) emphasize the difficulty for individuals to navigate and access services because of the “silo” approach in the healthcare system (55). *Ontario's Action Plan for Health Care* (2012) stressed that “patient centered integration is the right thing to do for patients, and for our healthcare system” (56). In Quebec, Fleury et al. (57) evaluated implementation strategies in the Quebec mental health reform that sought to improve accessibility, quality and continuity of care by “developing primary care and optimizing integrated service networks”. The authors recommend mental health reform focus on the development of network integration strategies and not solely on service implementation. The improvement of networks requires the implementation of more formalized integration strategies to better incorporate the continuum of care for clients with mental health disorders.

Vallerand and McLennan (33) surveyed child mental health agencies across Canada about the strategies they use to manage service demands. Collaborating with other providers and agencies was the most common approach that aligned with the Mental Health Commission of Canada recommendation for collaborative care or shared service model with the potential to improve access to services. Centralizing the intake process and using a triage system to prioritize care were found to reduce wait times for mental health services.

#### Community Level of Care

A comparative study found that unmet service needs in Canada were greater among individuals over age 65, those with low levels of education and rural residence (47). Fleury et al. (57) discuss the transition of individuals from mental health institutions to the community in a longitudinal study of 204 individuals with severe mental disorders (SMD), who faced isolation as they were integrated back into the community. They hypothesized a correlation between isolation and lower perceived adequacy of help received which consequently affects familial and community relationships (53). In order to better foster community integration for individuals with SMDs, supported employment, education, community treatment, and intensive case management were recommended.

Attitudes about mental health services and demographic variables predict service utilization. Individuals in socio-economically deprived communities have higher levels of depressive symptoms (37). Prevalence of mental health issues are also higher among individuals exposed to violence, crime, or imprisonment. Fleury et al. (37) assessed variables associated with health service use by individuals diagnosed with mental health disorders in Montreal. Individuals who used MHSU services had a worse perception of their mental health and lower life satisfaction compared to those who did not use mental health services (37). Predictors of service use were classified as: predisposing, enabling, and needs-related factors. Predisposing factors greatly hinder service use where females and those with higher education are more likely to seek health services in comparison to their community counterparts. The Canadian Health Act (1984) incents provinces to publicly fund services provided in hospitals and by physicians; availability of community-based services thus varies significantly with psychological services predominantly unavailable unless provided by a physician, to which access is limited. In comparison, community-based care is covered for individuals eligible for public health insurance under Medicare and Medicaid in the United States (47). International comparisons may further inform the implementation of health systems as they relate to access and utilization.

#### Youth and Families

An estimated 4.4% of Canadians aged 15 and older have a substance-use disorder, with alcohol dependence most prevalent at 3.2% (19). As integrated care is complex and little is known about what it entails for youth, designing, and implementing programs for youth lends further complexity. Settipani et al. (30) found an absence of comprehensive reviews of integrated care for youth MHSU in community settings. Their scoping review protocol proposes to better conceptualize integrated care for youth (populations, setting, service providers, interventions, infrastructure, coordination methods) and to identify constructs measured and evaluated. Although community-based integrated care hubs for youth with MHSU issues have emerged internationally, their key components require greater clarification. The Mental Health Commission of Canada released the *Evergreen: Child and Youth Framework for Canada* report (58), in which the use of “best available evidence” to inform treatments was a core value in the reform of Canada's publicly funded children's mental health care system.

Hunsley et al. (25) surveyed Canadian psychologists who care for children and youth in order to profile the services offered. Health services are provided in a wide range of publicly funded agencies and independent practices to patients from ethnically and socioeconomically diverse communities. Patients who received psychological services were found to be treated for the same problem by numerous sources, making coordination of their services an important issue. The majority of psychological practitioners (68%) reported providing more than one half of their services in a public practice context (e.g., health care facility, school board), while one third provided more than one half of their services in an independent practice context (25). Considerable evidence stresses the mental health problems of children and adolescents can foster lifelong behaviors. Hunsley et al. (25) recommend psychologists examine and possibly alter the relative balance of child- and adult-oriented training options to ensure availability of well-trained psychologists to deliver child and youth services (25).

Séguin et al. (18) identified the personal and social circumstances of suicides in New Brunswick to address the service needs of individuals and their families and improve suicide prevention by using direct proxy-based interviews, medical chart reviews and telephone contact with informants. Of the 109 suicide deaths identified, 42% of individuals had concurrent mood and substance use disorders with long-term destructive repercussions on individuals, family members and their social circles (18).

The Ontario Youth Wellness Hubs (YWHO) are being piloted to improve service standards and models of care for youth aged 12–25. The goals include increased access to timely, integrated mental health and addictions services for adolescents and transition-aged youth. Elements of the model include evidence-based services matched to individual need, co-located MHSU and primary care, improved holistic care, system functioning and mental well-ness outcomes (59). Funding of $3 million divided across multiple sites for 3 years supported the transition to offering integrated and co-located MHSU services. ACCESS Open Minds is a similar model being piloted across several provinces supported by the Canadian Institutes of Health Research and the Graham Boeckh Foundation (60).

#### Concurrent Early Psychosis and Substance Use Care

Despite an expansion of Early Psychosis Intervention (EPI) programs for youth in Ontario, a retrospective cohort study by Kozloff et al. (34) found 40% of youth with psychosis discharged to the community from their first emergency department (ED) visit received no outpatient mental healthcare within 30 days and over one in 10 received no care by 1 year. As EPI Programs in Canada receive a significant proportion of their referrals from EDs, better care coordination between EDs and EPI Programs was advised. Nolin et al.'s (35) survey of EPI programs in Canada found they all accepted patients with a concurrent substance use disorder. Nine of the 11 EPI programs offer services that address substance-induced psychosis (35). The same proportion use a case management model of care in which one clinician (occupational therapist, social worker, nurse) combines service delivery with coordination in which access to related services is brokered (35).

#### Homelessness

An estimated 35,000 people are homeless on any given night and 235,000 experience homelessness over a year in Canada (36). A significant proportion of homeless individuals suffer from substance dependence and concurrent mental illness (48). A longitudinal study on the vulnerably housed and homeless found more than half (52%) report a past diagnosis of a mental health problem (61). Latimer et al. (36) determined the annual costs of homelessness from a societal perspective, and individual characteristics associated with higher costs. A Housing First support program (At Home/Chez Soi) trial offered people access to permanent housing with long term support or usual treatment for those with mental health disorders (in Vancouver, Winnipeg, Toronto, Montreal, and Moncton) from 2009 to 2011. Given restrictions in sharing administrative data between province, Canadian Institute of Health Information data was used to estimate the costs of physician services, hospital stays, outpatient, and emergency department visits. The costs for people who were homeless longer tend to be higher (36).

Fleury et al. (37) found the ratio of renters to homeowners was a predictor of service utilization, that led them to advise that neighborhoods with a high proportion of rental accommodations be targeted as a public health priority to improve mental health and service use, as the needs of individuals facing homelessness tend to be neglected. McMain (17) argues that key indicators of severity be assessed including criminal justice system involvement, lack of productive activity, sexually risky behaviors, high healthcare use, early onset of substance use, polydrug dependence and low self-efficacy to resist psychoactive substance. Among users of homeless shelters in Toronto, lifetime diagnosis of mental illness or substance use was found to be as high as 67% and 68%, respectively (48).

The goals of Housing First services include reduction of unnecessary hospitalization and ED visits (48). Although numerous studies observed a positive outcome with Housing First for individuals with concurrent disorders, others found no difference in healthcare use between program participant and control groups (48). A study that examined whether substance dependence predicted healthcare use among participants in Housing First trials found the average number of ED visits was 4.2 per person, per year (48). Several studies link Housing First to increased residential stability and reduced health service use, particularly for those with the most serious illnesses, while others found no significant correlation. Cheung et al. (48) hypothesize this may be due to sample differences in terms of higher burden of medical and psychiatric comorbidities, severity of substance use and healthcare systems across jurisdictions that varied in terms of service coordination (40).

### Integration and Coordination of Care

Coordination and integration are closely related concepts. Coordinated care involves “actively managing all elements of the continuum of health and care services required by individuals and communities in order to achieve a seamless care pathway for the individual or client group” (46). Integration alternatively aligns collaboration between diverse providers through organizational, administrative, service delivery, clinical and funding approaches to support continuity of care by linking patient care across professional, organizational and system boundaries to improve efficiency (62). Integration can thus be considered as both a *process* that systematically arranges patient care across professionals and organizations; and integrated care as an *outcome* of the patient experience. Of four types of integration (organizational, functional, service, and clinical), *organizational* integration brings several organizations together through mergers or coordinated provider networks. *Functional* integration involves integrating administrative functions through for example shared electronic patient records. *Service* integration connects different clinical services at an organizational level through multidisciplinary teams for example. *Clinical* integration fosters a single, coherent care process by using shared guidelines (62).

Issues concerning integration were addressed by the report *Respect, Recovery, Resilience: Recommendations for Ontario's Mental Health and Addiction Strategy* (2010) that emphasized better coordination across the health system would help reduce avoidable ED visits and long waits for mental health and addiction services (63). Health Canada's report *Best Practices for Concurrent Mental Health and Substance Use Disorders* advised knowledge dissemination of best practices related to approaches for integration of concurrent disorders services (64). Although coordinated and centralized models of care exist throughout Ontario, they remain underdeveloped and precarious (46). ConnexOntario is an example of a centralized program that facilitates access to treatment and support services with varying levels of collaboration with regionally-accessed services (46).

#### Governance of Coordinated Care

Wiktorowicz et al. (22) compared models of governance that support coordination in 10 mental health networks across Canada. Networks were defined as a set of organizations and the relations among them that serve as channels through which communication, referrals, and resources flow (22). Networks develop efficient programs of care by coordinating primary, secondary, tertiary and community health and social services to simplify access for patients (22). Networks were categorized into one of three models of inter-organizational coordination: *mutual adjustment* based on voluntary exchanges (e.g., client referrals) between organizations without a formal mechanism of coordination (22); a *corporate* structure in which a regional authority integrates management of care (e.g., through oversight of psychiatric hospitals and community mental health centers), while in an *alliance* autonomous organizations form a coalition (22). Coordination was not well-supported when four aspects were considered: (1) Budget and planning decisions made at different jurisdictional levels (provincial vs. local level); the divided authority meant that organizations that reported to the Ministry were not held accountable when their services were not aligned with agencies in their network; (2) Hospitals had few incentives to align their care with community services, leading to delays in care for patients returning to the community who were more likely to “fall through the cracks” and be re-hospitalized; (3) Insufficient resources to develop information systems and electronic platforms that foster coordination; (4) Developing trust and cooperation among a large number of organizations in a metropolitan context could pose a challenge and require a regional strategy (22). Rush and Saini (46) suggest the lack of provincial description of available services and published syntheses of relevant research limits the evolution and evaluation of coordinated models of care.

Implementation of integrated MHSU service networks was found to rely on key strategies. Fleury et al.'s research based on Quebec's mental health reform (2005–2015) found that regional networks with strong governance and diversified resources supported better coordination of care and patient outcomes (51). Simplifying formal procedures such as the sharing of clinical records and referrals within and between organizations was found to promote collaboration and continuity of care between primary and specialized mental health services (57). Shared- or cross-training also supported service integration (19, 57) through knowledge translation on concurrent MHSU intervention methods (65). Facilitating knowledge sharing and collaboration between frontline and primary care providers was found to reduce harm and avoidable death, especially in the midst of a rising opioid epidemic. Knowledge sharing was also associated with enhanced perceived work role performance (66) and promoted shared vision and practice for integrated care among providers, reducing discrepancies in care (57).

Henderson et al. (42) demonstrated the positive outcomes of Integrated Collaborative Care Teams (ICCT) for youth programs in three Toronto neighborhoods. ICCTs include co-located MHSU care providers (e.g., youth workers, social worker, psychiatrist, nurse practitioner); trained peer support workers; access to a primary care provider and a care navigator responsible for coordinating care among the various specialists (42).

#### Competing Concepts for Concurrent Disorder Care

A shift is needed in how MHSU treatment is conceptualized, organized, and funded (21). System integration will enhance system efficiency and effectiveness, minimize program and administrative duplication, and reduce the likelihood of clients being misdirected, misdiagnosed or lost in the system (21). Some however, argue that full integration is unnecessary and enhanced co-operation, co-ordination and communication between agencies as an alternative (21). In a series of research interviews, an advocacy organization stated, “You can't have expertise in everything in one place; I actually think that we fail when we do that” (21). Integrated care should be more widely provided as individuals with concurrent disorders often experience a spectrum of health and socioeconomic issues including housing instability and justice system involvement. Selick and Wiktorowicz (21) conclude there are two worlds in addictions: professional staff and peer workers. And there are three worlds in mental health: psychiatry, which is focused on medication; community services, which are more psychosocially oriented; and the self-help consumer movement. In order to achieve integrated care, all of these fundamental components must align.

Torchalla et al. (40) examined the evidence on psychotherapeutic integrated treatment (IT) programs for individuals with concurrent disorders and trauma histories. Through their systemic review of 17 trials of integrated treatment programs, the majority reported that they effectively reduced PTSD and substance use disorder (SUD) symptoms over time (40). Similarly, Brousselle et al. (23) identified key factors required in integrated services and treatments for patients with concurrent disorders. Their study suggests integrated care be more flexible, to adapt the process of integration to a client's particular context. Vasiliadis et al.'s (39) analysis of self-reported MHSU service use found that of those with suicidal ideation and drug dependency, only 44.1 and 37.3% sought services. Of those for whom alcohol or illicit drug dependency interfered with daily life, only 26 and 27%, respectively sought care (39). Brousselle et al. (23) conclude that patient characteristics will ultimately drive the reorganization of the patient care experience.

#### Supports for Integrated Care

In Quebec, Fleury et al. (51) evaluated a pilot project to establish an integrated service network for adults with severe mental disorders in an urban area. The study was designed to offer support for decision-making, and solve problems as they arise. In focusing on the network of services provided and the organizations that make up the networks, the inter-organizational relationships fostered aided in improving quality of care. The first stage of implementation consolidated the range of resources available within the network. The integration strategies advanced by the authors validated the ability to transform the healthcare system based on its existing structures and resources while allowing for gradual change within the services.

McKee's (29) research sought to drive the reform of the mental healthcare system further and bridge the gap to treatment. The study suggested the implementation of change leaders as the system branches into integrated care. Change leaders need to be embedded within the program and remain long after initial integration to provide ongoing clinical supervision and model the novel, unified treatment philosophy (29). By including post treatment integration plans, programs are more likely sustainable and encompass the holistic needs of individuals with concurrent disorders.

#### Scope of Practice

Important sub-themes for integration and coordination were scope of practice and gaps in care. As primary care practices (PCPs) prescribe between 60 and 80% of psychotropic medications they play an important role in integrated care, where the same team treats physical and mental health problems and can achieve improved depression outcomes. Receiving services from PCPs can be less stigmatizing, more coordinated and more accessible than mental health specialist services. Ontario employees who received integrated care had fewer short-term disability days and returned to work faster. Integrated care was associated with shorter referral delays, reduced time in treatment, fewer appointments and lower treatment costs and addressed both mental health and substance use needs, which is important given the high rates of concurrent disorders (67). As the management of substance use involves screening, assessment and intervention, whose role it is to administer the screening tool, the quality of the tool and time involved are issues. While treatment should follow assessment, identifying relevant services and making referrals can be time consuming. PCPs may have difficulty recruiting addiction specialists and physicians with prescribing licenses to administer methadone for example (67).

Medical school and continuing education on substance use is considered inadequate, inconsistently applied and a low priority. Physicians cite a lack of confidence in their ability to offer these services. As the optimal management of many addictions (e.g., opioid dependence) involves a combination of pharmacological strategies and psychotherapeutic interventions that can increase the time needed for communication, physician practices may be deterred from offering them.

Although physicians engaged in integrated care express concerns about liability insurance issues, the Canadian Medical Protective Association (CMPA) that provides liability protection argues that while fear of increased medico-legal liability is cited as a barrier to health professionals working collaboratively, there is no need for extensive changes to the medical liability system (31). They emphasize the importance of both physicians and non-physician providers working collaboratively to attain professional liability protection and/or insurance coverage. The CMPA stresses this issue should not impede integrated care (Physicians acquire liability protection through the CMPA; non-physicians are covered through professional liability plans purchased through the Family Health Team in Ontario for example). The benefits of greater satisfaction with the quality of care delivered and improved patient outcomes are important incentives for physicians to engage in integrated care (31).

Kurdyak et al. (38) assessed the relationship among psychiatrist supply, practice patterns and access to psychiatrists in Ontario Local Health Integration Networks (LHINs). As the supply of psychiatrists increased, outpatient panel size decreased for full-time psychiatrists (38). In Toronto, psychiatrists had 58% smaller outpatient and inpatient panels and saw 57% fewer new patients relative to LHINs with the lowest psychiatrist supply (38). Concerningly, 10% of full-time psychiatrists in Toronto saw fewer than 40 unique patients and 40% saw fewer than 100 unique patients annually (38). In LHINs with lower supply the proportions were around 4 and 10% respectively (38).

#### Collaborative Care

Although models of collaborative care vary, they enable MHSU and primary care providers to work together more effectively to improve care. A position paper by Kates et al. (32) identifies common components of collaborative care to include use of a case manager to coordinate care, access to psychiatric consultation, evidence-based treatment guidelines, skill enhancement for primary care providers and access to psychological therapies. The benefits include better clinical outcomes, more efficient use of resources and improved access to care (32).

Assertive Community Treatment (ACT) offers an alternative model in which an inter-disciplinary team of 10–12 practitioners accept shared responsibility to offer care to a caseload of 60–100 patients. Instead of coordinating services across agencies, the team delivers services directly in community settings to facilitate in crisis support, treatment and rehabilitation. Treatment populations are generally among the most seriously ill, including those with concurrent disorders and those who have been committed to community treatment (68). As ACT programs are expensive, access to them is limited that can lead to gaps in care.

#### Gaps in Care

The historical separation of substance-use from mental health services has limited health professionals' scope of practice for concurrent disorders and produced gaps in care. Psychological services available to children are limited (25). The Mental Health Commission of Canada (MHCC) found effective MHSU care requires collaboration among service providers, service users and their families (69). While over half of patients are referred by a psychologist to another professional or agency; the services are not necessarily coordinated and collaborative (69).

The National Psychiatry Waiting List Survey indicated the average wait-time for “urgent referrals” made by family physicians to psychiatrists was 2-weeks, while the wait-time for “elective referrals” was 7-weeks (70). The ICCT approach for youth reduces wait-times and produces more youth- and family-friendly services to complement existing services (42). Denomme and Benhanoh (41) found that family member-oriented treatment programs led to increased participant knowledge of substance-use and concurrent disorders and resulted in better coping capabilities among families.

#### Referral to Psychotherapy

In Quebec, Roberge et al. (49) examined access to psychotherapy for anxiety disorders in a sample of primary care patients who met DSM-IV criteria for panic-, generalized anxiety- or social anxiety disorders. They found 40% reported seeing a psychologist in the past year and more than a third reported being referred to a psychologist by a primary care physician, which is not included in publicly insured healthcare (49). There are twice as many psychologists per capita in Quebec compared to other provinces. Lack of resources and poor collaboration between family physicians and psychologists limits access to evidence based psychological treatment. Bradley and Drapeau (50) found that 77% of psychologists and psychotherapists (*N* = 1,275) practicing in Quebec advocated for greater accessibility to publicly funded psychotherapy (50). Additional concerns among the sample involved expanding the scope of referrals, instilling greater supervision and including employment assistance with psychotherapy treatment (50).

In New Brunswick, Talbot et al. (26) surveyed family physicians (*n* = 152) on their treatment and referral practice for adults with significant anxiety or depressive symptoms. Low referral rates were found; 61% of physicians treat over 50% of anxiety or depressive patient symptoms in their practice (26). These findings suggest that many patients do not access psychotherapy. Addressing the attitudes of family physicians and stressing the necessity of integrated care and collaboration of various health professionals would help close the gap of unmet mental health needs.

### Policy

Integration and coordination of care is multifaceted as it involves translating policy into programs that encompass numerous stakeholders and agencies. MHSU care thus entails multilevel governance involving coordination of provincial, regional and sub-regional health and social services (22). Provincial Ministries of Health are responsible for mental health policy including aligning policies with the Ministries of Social Services, Justice etc., whereas regional health authorities oversee program operation including the development of regional and sub-regional service integration networks and liaising with primary care and other physician practices. Changes in provincial and regional governance affect the organization of and incentives that support service coordination. Within Canada's highly decentralized federal system, healthcare may be the most politically contested policy arena, resulting in intensely political policy negotiations between federal, provincial, and territorial governments as well as physician associations (45).

#### Accountability and Monitoring

In the absence of targeted federal transfers and effective accountability mechanisms, history reveals little hope for provincial and territorial governments to expand MHSU care on their own (45). A review of Auditor General reports across provinces from 2000 to 2018 reinforced the thematic challenges that emerged in the scoping review concerning access to care, service coordination and policy that prevent concurrent MHSU service needs from being addressed. The Auditor General of British Columbia found few specialized services for concurrent MHSU disorders and the situation is worse for rural and hard-to-reach populations (71). For instance, Northern Health which provides healthcare to rural and remote communities in BC with a high proportion of Aboriginal people has the highest rates of concurrent disorders (71). Low income neighborhoods in Quebec and BC were found to have a higher prevalence of unmet MHSU needs (43, 71). Although psychotherapy is an effective intervention for concurrent disorders (72), access is limited. Unlike Australia and the United Kingdom where psychological services are publicly accessible, most services in Canada are accessible to individuals with employer-based health insurance plans which has inadvertently instilled a two-tiered system of care (73, 74).

#### Policies Concerning Access to Care

Youth and adult access to concurrent disorders care was found to be limited. Until recently, Nova Scotia lacked a wait-time standard for child and adolescent mental health and substance-use services (75). Even where waitlist standards are mandated, it is not uncommon for youth and adults seeking inpatient mental healthcare or detox services to be turned away (76, 77). The service access issue is compounded by the fact that over one-third of individuals with concurrent needs miss appointments or treatments (77). Although stable housing can improve health outcomes for people with concurrent disorders (78, 79), many provinces lack information on the demand for supportive housing nor the units available to those in need (71, 80, 81).

Although physicians are usually the first point of contact for people with concurrent disorders, their care is not well-integrated with community-based healthcare services (81). Resultantly, transitions between specialty, acute, and community-based care are not well-managed making the system difficult for patients with complex MHSU needs to navigate (81–83). While community-based services (i.e., counseling, drug therapy and social housing) can be more effective and cost-efficient than hospital-based care (84), they are at capacity with long wait lists (71, 75, 80, 81). And although 10% of psychiatric hospital patients in Ontario were found to no longer require specialty care, a lack of supportive housing prevented their discharge (76).

Improving public access to psychological therapies can reduce bottlenecks in care by enabling psychiatrists to spend less time administering psychotherapy and more time seeing patients. Findings on psychiatrist supply and practice patterns in Ontario indicate that increasing psychiatric supply and duration of psychotherapy do not necessarily improve access to psychiatric services (38). Lack of coordination and access to services increases vulnerability to homelessness, hospital readmission and incarceration. Gaps between hospital and community-based care also leads to higher rates of hospital readmission (83, 85), referred to as the *revolving-door* syndrome. There is also a strong link between psychiatric deinstitutionalization and the overrepresentation of populations with MHSU needs in the criminal justice system, referred to as *trans-institutionalization*. Inmates in correctional facilities have a disproportionately high occurrence of MHSU issues and those with MHSU issues are more likely to be reconvicted (86, 87). Given a lack of clarity on which entity is responsible for providing concurrent MHSU services in provincial correctional institutions, gaps in accountability were found (86).

#### Policy Progress

Accountability and monitoring of policy progress in coordinating access to care reflects a further disconnect. Since the deinstitutionalization of psychiatric hospitals (beginning in the 1960s), jurisdictions shifted away from institutions and decentralized mental healthcare (71). In the past two decades, provinces reformed their MHSU systems to improve access and continuity of care through integrated service networks and shared care teams (57, 72, 75, 88). Interdisciplinary care models can take many forms (i.e., ACT teams, Intensive Case Management teams, Family Health Teams) and are usually provided through regional health authorities and contracted services providers (71, 88). While interdisciplinary care teams can reduce healthcare costs, the current fee-for-service funding model limits participation of physicians and other primary healthcare providers (81).

The Ontario *Patients First Act* (2016) and amendments to the *Local Health System Integration Act* (2006) equipped LHINs to better integrate healthcare systems by expanding the scope of a “health-service provider” under a LHIN to include Family Health Teams (88). Although LHINs now have a greater role in managing primary care, accountability for primary-care physicians remains under Ministry responsibility (88). An audit of mental health services in Alberta in 2015 found a disconnect between MHSU community providers and family physician practices; “half of all primary care networks had no mental health providers and seven had less than one full-time equivalent” (81). The divide between MHSU healthcare providers complicates sharing of patient information; providers do not share information even where there are no legal barriers (81).

#### Monitoring Policy Implementation

Provinces were also found to lack a governance mechanism to monitor the progress of policy implementation (75). Despite implementing strategies to improve access to MHSU care, many provinces do not have an effective process for monitoring provider performance, payment nor the ability to manage the capacity and demand for service (71, 75, 77, 81, 88). Bartram and Lurie (45) suggest expanding the mandate of the Mental Health Commission of Canada to include a monitoring function, or entrusting monitoring to provincial and territorial auditor generals as per the recommendation of the MHCC report *Out of the Shadows at Last*. Provincial auditor generals have attributed service gaps in the delivery of MHSU care to historical funding patterns that ignore assessed needs (71, 81, 88, 89). Service gaps result in people receiving different levels of care despite similar needs that widened MHSU inequities (80).

#### Funding

While the Canada Health Act (1984) could have addressed the growing need for broader health insurance by making coverage for non-physician health and mental healthcare providers such as nurses and psychologists a condition of federal funding transfers, instead it only addressed the issue of extra billing. The MHCC (69) recognized the importance of investing in mental health and social spending to strengthen the capacity of the MHSU system as part of the Mental Health Strategy for Canada. The current share of health spending on mental health in Canada at 7% (90), pales in comparison to other high income countries that spend up to 18% on mental health, with the United Kingdom spending 13% (91). For example, Ontario made new investments of $16.45 per capita in mental health compared to investments of $62.22 per capita in the U.K. and $98.13 in Australia between 2004 and 2011 (92). During this same time, Ontario invested $220 million in community mental health services and $18.5 billion in health care (92). Ontario invested an additional $180 million in community mental health services and $3.8 billion in health care from 2011 to 2016 (93).

The Canadian Institute for Health Information (2015) estimates that public funding from government transfers would need to increase to $3.1 billion per year, with an incremental base funding increase of $310 million each year, to close the MHSU gap and increase the share of mental health spending from seven to nine percent, with additional investments in social spending as the National Mental Health Strategy advised (69, 94). The MHCC (95) commissioned report *Making the Case for Investing in Mental Health* anticipated that such investments would reduce MHSU expenditures by over $179 billion and reduce employer expenditures due to productivity loss by $76.1 billion. Without increased investment, the MHCC (95) predicts that the cumulative cost of MHSU issues to the economy will exceed $2.5 trillion by 2041.

With $5 billion in new federal funding to improve access to mental health services set to roll out over the next 10 years through the 2017 Health Accord, the window of opportunity could begin to close the long-standing gap in mental health funding. Bartram's analysis of potential accountability mechanisms advises tying targeted federal funds to specific initiatives, population groups or levels of evidence (45, 94).

## Discussion and Conclusion

An analysis of the literature on emergent models of service across Canada revealed that individuals with concurrent disorders present with complex needs that are often difficult to treat (96). A major challenge in addressing concurrent disorders has been integrating services between MHSU providers and community agencies. The independent development of mental health and substance-use services made accessing appropriate care more difficult and led to greater mental healthcare need among individuals with concurrent disorders (4). Considerable research on improving system and service level integration has emerged in recent years. While collaborative care models can simultaneously address multiple needs (i.e., concurrent disorders), our findings highlight the barriers to access care that remain.

Two general approaches to achieve service-level integration were identified: co-located MHSU services or collaborative care models across MHSU service providers (72). Successful healthcare reform for concurrent MHSU disorders requires greater attention to develop and operationalize integrated care models and service networks. While the models of integrated MHSU care envisioned would improve coordination among providers and access to services, structural barriers inhibit implementation. Despite several pilot projects and frontline operational practices that support integrated MHSU care, governments and legislatures have yet to accept responsibility for healthcare restructuring (81). The Auditor General of Alberta (81) identifies three elements that must be incorporated into healthcare frameworks to achieve integrated care; (1) structuring the healthcare system to include clear responsibility of roles, internal frameworks for funding and accountability for results; (2) integration of physicians through financial incentives or alternative pay models; and (3) clinical information systems to ensure appropriate sharing of patient information. These elements are critical to facilitate partnerships for collaborative care and address service gaps in concurrent MHSU care.

Lessons can be drawn from the barriers to concurrent MHSU care found and evidence concerning successful implementation strategies for integrated care models and the service networks on which they rely. The findings suggest formal network decision-making supports, implementation strategies and accountability mechanisms that monitor progress in implementing system reform would improve access to care. Despite limited progress made to integrate mental health with substance-use care, issues of policy accountability for system-level integration inhibit the delivery of coordinated care. Greater attention to operationalize and implement integrated service networks to address unmet MHSU needs and related harms requires changes to the funding and structure of provincial MHSU healthcare systems. At the governance level, funding incentives for providers should better align with the care needs of patients.

A paradigm shift in which providers are seen as an integral part of a broader system of collaborative MHSU care reflects the future. This entails supporting providers to be part of a team, educating them on the opportunities to refer patients to related care providers such as early psychosis and substance use therapy, as well as an openness to accept referrals from community-based providers to better meet patient needs (97). This could include engagement in networks that offer centralized patient intake and care referral, and enabling primary care practices to include allied health professionals such as case managers to better serve those with concurrent disorders. Reforming payment modalities can reward physicians for their involvement in collaborative care teams and local networks of centralized care access. Legislative changes can also improve the scope of concurrent MHSU care by including more psychologists and front-line workers under the fee-for-service payment system. Sharing clinical information among providers enhances collaboration and better patient outcomes. Future studies on integrated networks with centralized service access that include cross-training mechanisms to foster common understandings, relationship-building and reduce discrepancies in care would enhance our understanding of promising models of care.

## Limitations

Our assessment of the challenges in accessing coordinated MHSU care included a broad range of literature that was triangulated with an analysis of auditor general reports to counter potential bias. A number of limitations nonetheless remain. First, the review was limited to the search of English language studies even though research on coordinating MHSU care has been conducted in French speaking provinces. Moreover, the analysis may be somewhat unbalanced given the number of studies based in Ontario. Although auditor general reports from Alberta and Ontario were most referenced, findings from many other provinces were included (B.C., Nova Scotia, New Brunswick, Newfoundland and Labrador, and Saskatchewan). The results may also not be generalizable outside Canada as the design of healthcare systems varies across countries (i.e., public vs. private funding). Lastly, despite our attempt to conduct a thorough literature search, it is possible we missed studies on models of MHSU care in our analysis.

## Author Contributions

MW conceptualized the study and introduction, contributed to the analysis of the literature and auditor general reports, and substantively edited the manuscript. AA conducted the literature search and analysis, wrote the methods, developed the decision tree, table summarizing the studies included and first draft of the results. KD analyzed the auditor general reports, contributed to the analysis of the literature, developed the discussion and conclusion and organized the references. SB contributed to the conceptualization of the study, supported the literature search, decision tree, and critically edited the manuscript.

### Conflict of Interest Statement

The authors declare that the research was conducted in the absence of any commercial or financial relationships that could be construed as a potential conflict of interest.
